# Introducing Summative Progress Testing in Radiology Residency: Little Change in Residents’ Test Results After Transitioning from Formative Progress Testing

**DOI:** 10.1007/s40670-020-00977-2

**Published:** 2020-05-13

**Authors:** D. R. Rutgers, J. P. J. van Schaik, C. L. J. J. Kruitwagen, C. Haaring, W. van Lankeren, A. F. van Raamt, O. ten Cate

**Affiliations:** 1 Department of Radiology, University Medical Center, Utrecht University, Heidelberglaan 100, 3584 CX Utrecht, The Netherlands; 2 Examination Committee of the Radiological Society of the Netherlands, Utrecht, The Netherlands; 3 Julius Center, Department of Biostatistics, University Medical Center, Utrecht University, Utrecht, The Netherlands; 4 Department of Radiology, Erasmus MC, Rotterdam, The Netherlands; 5 Radiological Society of the Netherlands, Utrecht, The Netherlands; 6 Department of Radiology, Gelre Hospital, Apeldoorn, The Netherlands; 7 Center for Research and Development of Education, University Medical Center, Utrecht University, Utrecht, The Netherlands

**Keywords:** Progress testing, Educational measurement, Internship and residency, Radiology

## Abstract

**Introduction:**

Educational effects of transitioning from formative to summative progress testing are unclear. Our purpose was to investigate whether such transitioning in radiology residency is associated with a change in progress test results.

**Methods:**

We investigated a national cohort of radiology residents (*N* > 300) who were semi-annually assessed through a mandatory progress test. Until 2014, this test was purely formative for all residents, but in 2014/2015, it was transitioned (as part of a national radiology residency program revision) to include a summative pass requirement for new residents. In 7 posttransitioning tests in 2015–2019, including summatively and formatively tested residents who followed the revised and pre-transitioning residency program, respectively, we assessed residents’ relative test scores and percentage of residents that reached pass standards.

**Results:**

Due to our educational setting, most posttransitioning tests had no residents in the summative condition in postgraduate year 4–5, nor residents in the formative condition in year 0.5–2. Across the 7 tests, relative test scores in postgraduate year 1–3 of the summative resident group and year 3.5–4.5 of the formative group differed significantly (*p* < 0.01 and *p* < 0.05, respectively, Kruskal-Wallis test). However, scores fluctuated without consistent time trends and without consistent differences between both resident groups. Percentage of residents reaching the pass standard did not differ significantly across tests or between groups.

**Discussion:**

Transitioning from formative to summative progress testing was associated with overall steady test results of the whole resident group in 4 post-transitioning years. We do not exclude that transitioning may have positive educational effects for resident subgroups.

## Introduction

Resident training becomes increasingly shaped into the molds of competency-based medical education (CBME) [1–5]. Through CBME, residents learn cardinal competencies to practice their specialty unsupervised. To decide whether residents master the required competences, programs use summative assessment tools [6, 7]. These can range from workplace evaluations to standardized skills tests and written knowledge assessments. Progress testing is a form of knowledge assessment that has gained ground in medical education in the past two decades [8–12]. It was originally developed in medical schools and later extended to postgraduate medical education [13–15]. A key feature of progress testing is the spaced repetition of comprehensive tests which intends to stimulate long-term knowledge retention [16].

Medical educators have often used progress testing as a purely formative tool [10, 12]. In recent years, however, a transition to summative progress testing has taken place in several institutions [17–19]. An important reason for this transitioning may be the need for accountability that weighs upon medical educators, as the aim of CBME is to demonstrate that graduating trainees meet professional standards [20]. When shifting to a different test format, educators should have an eye for the educational effects that assessment may induce [20, 21]. For example, summative assessment may constitute a stronger learning stimulus than formative assessment because trainees can potentially fail the former [22]. Following this, transitioning to summative progress testing may be expected to increase the knowledge level in participants. This presumed positive educational effect has been an additional reason for some program directors to transition from formative to summative progress testing in resident training [19]. In case transitioning does not induce a positive effect, then at least it should not impair the trainee’s knowledge level. Such a knowledge decrease is a genuine possibility since some research in undergraduates has suggested that education programs with summative progress testing may have lower participants’ test scores than programs with formative progress testing [23]. For postgraduate training programs, it is not known from the literature how residents’ knowledge levels develop after shifting to summative progress testing. This gap encouraged us to conduct the present study in which we investigated whether transitioning from formative to summative progress testing in a competency-based radiology residency program was associated with a change in participants’ test scores.

### Educational Setting

This study was carried out among Dutch radiology residents. Radiology residency in the Netherlands consists of a 5-year competency-based curriculum. New residents may enter residency throughout the whole year and residents may go through residency on a part-time basis which lengthens their training proportionally [24]. Residents are formatively and summatively assessed through various workplace-based assessment tools and written examinations, including a semi-annual comprehensive radiological knowledge test known as the Dutch Radiology Progress Test (DRPT) [25]. Residents are required to participate in the DRPT in all 5 postgraduate years (PGYs), establishing a total of 10 tests per resident, evenly distributed over PGYs 1–5. There is no concluding radiology board exam at the end of residency, but graduation has to be acknowledged by the national registration committee for medical specialists in order for the resident to register as a radiologist.

**Fig. 1:**
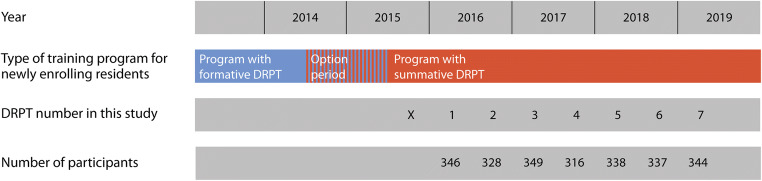
Change over time of the type of training program for newly enrolling residents and timing of the investigated tests of the Dutch Radiology Progress Test in this study. DRPT indicates Dutch Radiology Progress Test. In October 2015 (indicated by the x mark), the test failed due to technical reasons. July 2014–July 2015 was an option period during which newly enrolling residents were given the opportunity to choose (at the latest by June 2015) which of the 2 residency program variants they wanted to follow until graduation: either the former curriculum with an exclusively formative DRPT or the revised program with DRPT pass requirement

In 2003, the DRPT started as a purely formative test in the Dutch national radiology residency program [15]. In July 2014, this program was thoroughly revised by introducing radiological entrustable professional activities (EPAs) and by merging the training programs of radiology and nuclear medicine—which used to be separate residency programs—into 1 combined national radiology residency program. Moreover, this revision comprised transitioning the DRPT to include a summative pass requirement (defined below) with the aim to stimulate learning and to meet the need for accountability [19, 20]. As part of this transition, residents who started radiology training between July 2014 and July 2015 were given the opportunity to choose, at the latest by June 2015, which of the 2 program variants they wanted to follow until graduation: either the former curriculum with an exclusively formative DRPT or the revised program with DRPT pass requirement (see Fig. 1).

From July 2015 onward, all newly enrolling residents were required to follow the revised program. Residents who had started radiology training before July 2014 remained confined to the former curriculum. Following these regulations, from July 2015 onward, each resident in the DRPT was inextricably part of either of 2 non-randomized resident groups: (1) the group that followed the revised training program, including DRPT pass requirement (this we called the “summative resident group”), or (2) the group that followed the former curriculum, including an exclusively formative DRPT (this we called the “formative resident group”).

The DRPT’s summative pass/fail criterion was defined as follows [19]: residents must obtain a pass score for at least 3 individual tests that are taken in PGYs 2.5–5, while tests in PGYs 0–2.5 only serve formative purposes. Final year residents who are deemed to be at risk—based on their test results in PGYs 2.5–4—of failing to reach 3 pass scores by the end of residency are obliged to take (and pass) the examination for the European Diploma in Radiology (EDiR) of the European Society of Radiology before completion of residency. For these residents, the EDiR examination is considered an additional opportunity to demonstrate an adequate radiological knowledge level. If residents do not pass either of these summative criteria, registration as a radiologist in the Dutch medical register is postponed. These residents are allowed to re-sit for examinations until sufficient DRPT pass scores are achieved or until the EDiR is obtained, complying with the competency-based nature of the residency program. They can re-sit the regular DRPT tests as often as necessary at the usual semi-annual interval, but no extra tests are offered.

### Study Purpose

Our study had 2 purposes: first, to explore whether the introduction of summative progress testing was associated with a change in test scores in the summative resident group during a 4-year observation period; second, to investigate whether scores in the summative group differed from scores in the formative resident group during this period.

## Materials and Methods

### Dutch Radiology Progress Test

The DRPT is organized by the Examination Committee of the Radiological Society of the Netherlands [25]. All residents are required to participate, but in individual cases, residents may apply at the Examination Committee for dispensation from participation for various reasons, such as congress attendance, leaves, health issues, or other circumstances in personal life. In previous years, between 1 and 16% of residents received this dispensation per test administration [15]. For each test occasion, a new set of test items is drafted using a blue print. Various response formats are used, including true/false items, single right multiple choice items, drag-and-drop items, and long-list-menu items. The DRPT is administered digitally with software (http://vquest.bluefountain.nl/en/) that has been developed specifically for image-based testing [26]. After each test, items are reviewed in response to psychometric item analysis and written item feedback from participating residents, after which the Examination Committee decides on exclusion of unreliable or flawed items (generally, on average less than 10% of items per test [15]). Subsequently, the Examination Committee determines the final test scores for all participating residents. Test results are communicated to individual residents and program directors through individualized report forms that give the resident’s overall absolute test score, scores on radiological subdomains, and pass score when appropriate.

In the tests from April 2017 onward (test #3 in this study), the Examination Committee assessed—following the DRPT’s summative pass/fail criterion—whether PGY 2.5–5 residents in the summative curriculum had passed the test. For this, a pass standard setting method was used that combined a criterion- and a norm-referenced approach [27]. Before 2017, no test passes needed to be assessed because in these years the summative resident group consisted entirely of residents who had less than 2.5 training years, because residents enrolled in the summative curriculum only after July 2014. As tests in PGY < 2.5 merely serve formative purposes in the DRPT regulations, no test passes needed to be assessed before 2017. This time period included tests #1 and #2 in the present study. As opposed to PGY 2.5–5 residents in the summative curriculum, no actual test passes needed to be assessed for residents in the formative group, nor for PGY 0–2.5 residents in the summative group, because tests were merely formative for them. However, their test report form did specify whether their test score was equal to or higher than the pass standard in the test concerned.

### Data Collection

We reviewed all DRPTs that were taken from July 2015—when residents became inextricably part of either the summative or formative resident group—to April 2019. We chose for this 4-year observation period because its end was on the eve of the 5-year existence of the revised national radiology residency program (introduced in July 2014), while the former curriculum with an exclusively formative DRPT was discontinued. Graduation of the first residents who were trained according to the revised program (that included DRPT pass requirement) was expected shortly after the 4-year observation period, given the fact that radiology residency in the Netherlands nominally spans 5 training years. From an educational point-of-view, we found this an appropriate moment to review the revised DRPT format.

During our observation period, 7 tests were taken (see Fig. 1; the October 2015 test failed due to technical reasons). In each test, we assessed the number of training years of participating residents at the time of testing, rounded off to 1 decimal place. Using number-right scoring, we calculated per test the *absolute sum score* for each participating resident. As an example of absolute sum score calculation, consider a resident who would answer 121 items correctly in a given test. This would give an absolute sum score of 121. Subsequently, in order to compare residents’ test scores between separate DRPT tests, we calculated the *relative test score* for each participating resident per test. We found that absolute test scores were not suitable for this purpose for two reasons. First, the number of test items that was excluded from the initial 180 items after post-test analyses varied across tests, resulting in a varying number of items. This is a limitation when comparing absolute scores between tests, because a test with fewer items has lower absolute scores than a test with many included items, even though the residents’ knowledge level may be similar in both tests. Second, chance success scores (i.e., scores resulting from random guessing) varied across tests. Again, this is a limitation when comparing absolute scores between tests, because a test with a low chance success score will likely have lower absolute scores than a test with a high chance success score, even though residents may have similar knowledge levels in both tests. Because of these limitations, we calculated relative test scores, in which we accounted for inter-test differences in number of included test items and chance success scores using the following formula:

$$ \frac{a-c}{b-c}\times 100 $$

where *a* is the resident’s absolute sum score in a given test, *b* is the highest possible absolute sum score in that test, and *c* is the chance success sum score in that test. From this, it follows that *a* − *c* is the resident’s absolute-minus-chance success sum score and *b* − *c* is the highest possible absolute-minus-chance success sum score in the test concerned. We calculated the *chance success sum score* (*c*) by summing the chance success scores of all individual items in the test concerned, in which the chance success score of a given individual test item was calculated as the reciprocal of the number of answer options in that item. We rounded relative test scores off to 1 decimal place. As an example of relative test score calculation, consider test #1 (Table 1) in which the highest possible absolute sum score (*b*) was 173, because 7 of 180 items were excluded. The chance success sum score (*c*) in that test was 69, giving 104 (=173 − 69) as the highest possible absolute-minus-chance success sum score (*b* − *c*) in test #1. If a resident in that test would achieve an absolute sum score (*a*) of 121, this resident’s absolute-minus-chance success sum score (*a* − *c*) would be 52 (=121 − 69), giving a relative test score ([*a* − *c*/*b* − *c*] × 100) of 50 (i.e., 52/104 multiplied by 100). Relative test scores in a given test could run from 0 to 100, but could also be negative if a resident’s absolute sum score was lower than the chance success sum score.

**Table 1:** General test characteristics and overall test scores

	Dutch Radiology Progress Test						
	1	2	3	4	5	6	7
General test characteristics							
Date	Apr 2016	Oct 2016	Apr 2017	Oct 2017	Apr 2018	Oct 2018	Apr 2019
Participants (*n*)	346	328	349	316	338	337	344
Test items (*n*)	180	180	180	180	180	180	180
Excluded test items (*n*)	7	4	8	9	9	8	7
Highest possible absolute sum score	173	176	172	171	171	172	173
Chance success sum score	69	75	67	57	54	49	42
Highest possible absolute-minus-chance sum score	104	101	105	114	117	123	131
Pass standard (given as absolute-minus-chance success sum score)	47	44	48	58	53	61	66
Overall test scores (median (IQR))							
Absolute sum score	116 (21)	119 (23)	115 (20)	117 (21)	109 (24)	115 (30)	112 (28)
Absolute-minus-chance success sum score	47 (21)	44 (23)	48 (20)	60 (21)	55 (24)	66 (30)	70 (28)
Relative test score (max. 100)	45 (20)	44 (23)	46 (19)	53 (18)	47 (21)	54 (24)	53 (21)

*Apr*, April; *Oct*, October; *IQR*, interquartile range

In addition, for each test, we calculated the percentage of residents that had achieved a test result that was equal to or higher than the pass standard in that particular test. Such a test result signified an actual test pass for residents to whom the summative DRPT criterion applied (i.e., PGY 2.5–5 residents in the summative group). For residents to whom this criterion did not directly apply (i.e., PGY 0–2.5 residents in the summative group and all PGYs in the formative group), such a test result indicated that they had fictionally reached a test pass according to the summative DRPT criterion.

### Statistical Analysis

We investigated normality distribution of variables by visual inspection. We categorized residents in half-year cohorts based on the number of training years at the time of testing. In each of the 7 separate DRPTs, we calculated relative test scores per half PGY. We created a scatter plot of relative test score versus PGY in the summative and formative resident groups, pooling all 7 investigated DRPTs, to visually inspect whether there were overall differences between both groups in the increase of test scores over PGYs. To analyze differences in relative test scores across DRPTs, we performed the Kruskal-Wallis test. To analyze differences in relative test scores between summative and formative resident groups, we used the Mann-Whitney *U* test. We investigated differences in percentage of residents that achieved an actual or fictional pass score with the chi-square test. In each statistical test, we used the Bonferroni correction for multiple comparisons. A *p* value < 0.05 was considered statistically significant.

### Institutional Review Board Approval

The ethical review board of the Netherlands Association for Medical Education approved conduct of this study (dossier number 2018.7.8) and concluded that there would be no harm, deception, or disadvantage to subjects of the study, that autonomy of subjects was not compromised, and that informed consent was not necessary.

## Results

Table 1 shows general characteristics and overall test scores for the 7 separate DRPTs. The total number of participating residents per test varied from 316 to 349. In each test, 180 items were posed and per test between 4 and 9 items were excluded after post-examination test review. From tests #2 to #7, chance success sum score decreased and the highest possible absolute-minus-chance success sum score increased. The pass standard increased between most tests, which generally was related to the decrease of chance success sum score.

The distributions of absolute and relative test scores were negatively skewed when data of the 7 tests were pooled. In the 7 tests separately, absolute and relative test scores were negatively skewed in tests #2 to #7 and showed a bimodal distribution in test #1. Following this, we performed non-parametric statistical analysis of these variables.

**Fig. 2:**
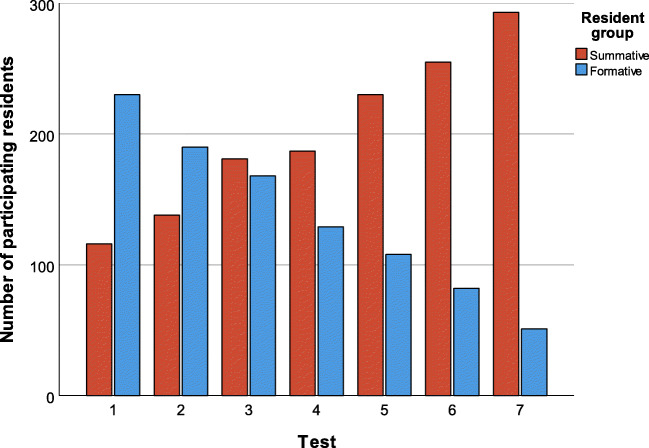
Number of participating residents in the summative and formative resident groups from tests #1 to #7

Figure 2 illustrates how from tests #1 to #7 the total number of residents in the summative group increased from 116 to 293 and in the formative resident group decreased from 230 to 51.

Table 2 shows the number of participating residents per PGY in the 7 tests. Due to our educational study setting, most tests had no residents in the summative condition in PGYs 4–5, nor residents in the formative condition in PGYs 0.5–2. Normally, residents would move to higher PGYs from tests #1 to #7. However, the rate at which this happens may vary between individual residents due to variations in their individual residency programs. These variations are explained by differences in part-time factors and number or duration of leaves taken. As a result of these variations, the number of residents per PGY varied between tests.

**Table 2:** Number of participating residents per postgraduate year

PGY	Group	Dutch Radiology Progress Test							
		1	2	3	4	5	6	7	Sum
0.5	S	43	35	45	20	37	32	48	260
	F	-	-	-	-	-	-	-	-
1.0	S	29	39	33	39	20	34	33	227
	F	-	-	-	-	-	-	-	-
1.5	S	33	31	40	35	39	24	29	231
	F	3	1	-	-	-	-	-	4
2.0	S	11	27	29	34	35	42	26	204
	F	23	4	1	-	-	-	-	28
2.5	S	-	6	27	32	39	34	39	177
	F	42	24	7	1	-	-	-	74
3.0	S	-	-	7	24	33	35	33	132
	F	32	45	23	10	6	2	-	118
3.5	S	-	-	-	3	24	33	36	96
	F	34	29	50	26	10	5	-	154
4.0	S	-	-	-	-	3	19	31	53
	F	26	26	35	42	29	13	8	179
4.5	S	-	-	-	-	-	2	16	18
	F	43	25	26	31	38	33	18	214
5.0	S	-	-	-	-	-	-	2	2
	F	27	36	26	19	25	29	25	187

*PGY*, postgraduate year; *S*, summative resident group; *F*, formative resident group

PGY 0.5 indicates training year 0–0.5; PGY 1.0, training year 0.5–1.0; PGY 1.5, training year 1.0–1.5; et cetera

**Fig. 3:**
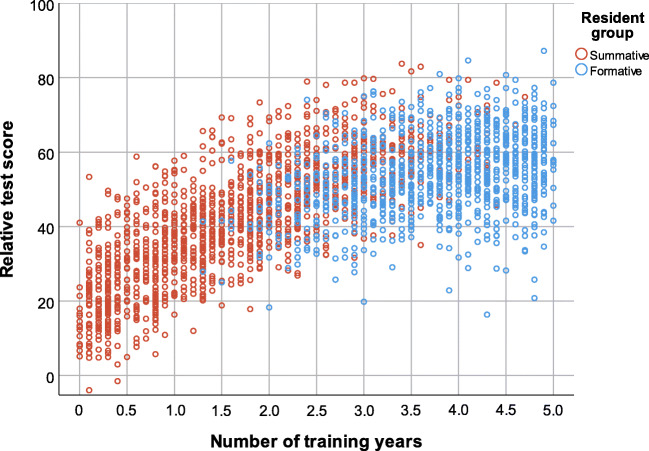
Scatter plot of relative test score versus number of resident training years, pooled for the 7 investigated progress tests

Figure 3 shows a scatter plot of number of training years versus relative test score, pooled for all participants from the 7 DRPT tests. Visually, the scatter plot of the summative resident group is congruent to the plot of the formative resident group. In addition, the scatterplot suggests that after PGY 3, individual outliers on the lower side of the plot are more frequently from the formative resident group than from the summative group.

Relative test scores per test and PGY are shown in Table 3. In the summative resident group, differences across tests #1 to #7 were not statistically significant in PGY 0.5, whereas in PGYs 1–3, these differences were statistically significant (*p* < 0.01, Kruskal-Wallis test). In PGYs 1–3, relative scores showed up and down fluctuations across tests #1 to #7 and we did not observe a consistent score trend over the course of tests. In later PGYs of the summative resident group, no significant differences across tests were found. In the formative resident group, differences across tests were statistically significant in PGYs 3.5–4.5 (*p* < 0.05, Kruskal-Wallis test), but not in other PGYs. Again, scores showed up and down fluctuations without a consistent score trend over tests. When combining results from all 7 tests (“overall” column in Table 3), residents in the summative group tended to have somewhat higher overall relative test scores per half PGY than those from the formative resident group. However, in none of the 7 tests separately, differences between the summative and formative resident groups were statistically significant in any half PGY, in case this included residents from the 2 groups. In both resident groups, overall relative test scores increased in roughly the first half of residency and flattened off in the second half.

**Table 3:** Relative test scores

PGY	Group	Dutch Radiology Progress Test								Kruskal-Wallis test across DRPTs #1 to #7
		1	2	3	4	5	6	7	Overall	
0.5	S	24 (12)	19 (15)	19 (16)	22 (19)	23 (11)	25 (10)	24 (16)	23 (14)	n.s.
	F	-	-	-	-	-	-	-	-	n.a.
1.0	S	33 (13)	26 (12)	32 (19)	32 (21)	31 (12)	33 (16)	36 (11)	32 (14)	*p* < 0.01
	F	-	-	-	-	-	-	-	-	n.a.
1.5	S	36 (9)	35 (14)	36 (10)	45 (13)	33 (14)	37 (13)	43 (9)	38 (12)	*p* < 0.01
	F	28 (-)	x	-	-	-	-	-	35 (16)	n.a.
2.0	S	45 (20)	39 (15)	44 (11)	49 (9)	41 (15)	47 (20)	43 (14)	45 (14)	*p* < 0.01
	F	44 (11)	37 (25)	x	-	-	-	-	43 (10)	n.s.
2.5	S	-	42 (22)	46 (12)	53 (12)	49 (13)	57 (11)	52 (19)	51 (15)	*p* < 0.01
	F	46 (14)	47 (10)	53 (18)	x	-	-	-	47 (15)	n.s.
3.0	S	-	-	49 (8)	56 (14)	50 (10)	61 (11)	61 (11)	56 (14)	*p* < 0.01
	F	50 (12)	50 (14)	50 (13)	61 (13)	49 (12)	54 (-)	-	50 (12)	n.s.
3.5	S	-	-	-	64 (-)	54 (14)	60 (10)	58 (7)	58 (10)	n.s.
	F	49 (15)	53 (14)	50 (15)	59 (12)	57 (20)	55 (16)	-	53 (15)	*p* < 0.05
4.0	S	-	-	-	-	48 (-)	64 (14)	60 (11)	60 (12)	n.s.
	F	57 (14)	56 (14)	53 (10)	60 (14)	54 (18)	63 (14)	65 (12)	57 (13)	*p* < 0.05
4.5	S	-	-	-	-	-	68 (-)	58 (14)	59 (14)	n.a.
	F	55 (16)	52 (14)	58 (11)	59 (12)	55 (16)	63 (13)	61 (17)	58 (15)	*p* < 0.05
5.0	S	-	-	-	-	-	-	71 (-)	71 (-)	n.a.
	F	58 (20)	53 (18)	58 (14)	58 (9)	57 (18)	58 (13)	60 (13)	58 (15)	n.s.

*PGY*, postgraduate year; *DRPT*, Dutch Radiology Progress Test; *S*, summative resident group; *F*, formative resident group; *n.s.*, not significant; *n.a.*, not applicable

PGY 0.5 indicates training year 0–0.5; PGY 1.0, training year 0.5–1.0; PGY 1.5, training year 1.0–1.5; et cetera

Data are presented as median with interquartile range (IQR) in parentheses. Subgroups designated by “x” consisted of 1 resident; no median and IQR are given in these subgroups

The percentage of residents that achieved a test score equal to or higher than the pass standard in individual DRPT tests is shown in Table 4. Both in the summative and the formative groups, differences across tests were not statistically significant for any of the half PGYs. When combining the results of all individual DRPT tests shown (“overall” column in Table 4), both the summative and formative resident groups showed that percentages increased in roughly the first half of residency and flattened off in the second half. Residents in the summative group tended to have somewhat higher overall percentages per half PGY than residents in the formative group. However, in none of the individual tests, differences between both resident groups were statistically significant in case a given half PGY included residents from the 2 groups.

**Table 4:** Percentage of residents per individual DRPT test that achieved a test score equal to or higher than the pass standard in that particular test

PGY	Group	Dutch Radiology Progress Test						Chi-square test across DRPTs #3 to #7
		3	4	5	6	7	Overall*	
0.5	S	2	0	3	0	0	1	n.s.
	F	-	-	-	-	-	-	n.a.
1.0	S	24	8	0	6	9	10	n.s.
	F	-	-	-	-	-	-	n.a.
1.5	S	13	23	21	4	14	16	n.s.
	F	-	-	-	-	-	-	n.a.
2.0	S	31	41	40	43	31	38	n.s.
	F	0	-	-	-	-	0	n.a.
2.5	S	59	59	59	79	51	61	n.s.
	F	57	100	-	-	-	63	n.s.
3.0	S	86	75	82	94	88	86	n.s.
	F	74	80	67	50	-	73	n.s.
3.5	S	-	100	83	85	94	89	n.s.
	F	74	89	80	100	-	80	n.s.
4.0	S	-	-	67	95	97	94	n.s.
	F	83	91	72	92	100	85	n.s.
4.5	S	-	-	-	100	94	94	n.s.
	F	96	86	84	88	83	88	n.s.
5.0	S	-	-	-	-	100	100	n.a.
	F	92	90	88	83	92	89	n.s.

*PGY*, postgraduate year; *DRPT*, Dutch Radiology Progress Test; *S*, summative resident group; *F*, formative resident group; *n.s.*, not significant; *n.a.*, not applicable

PGY 0.5 indicates training year 0–0.5; PGY 1.0, training year 0.5–1.0; PGY 1.5, training year 1.0–1.5; et cetera

*Overall refers to the overall passing percentage in DRPTs #3 to #7 and was calculated by pooling the numbers of residents from these separate DRPTs (both total number of residents and number that achieved the pass standard score in these tests)

## Discussion

The major finding of this study was that transitioning from formative to summative postgraduate progress testing was associated with little change in test results of the resident group in 4 years after transition. We found indications that residents from the summative group performed somewhat better than those from the formative group; however, no statistically significant differences between both groups were found.

Assessment and learning make an inseparable couple in medical education. Their relationship has changed over the years [21]. Traditionally, medical trainees learned by moving along a range of successive training blocks, each of them assessed by high-stake end-of-block examinations that were separated from the learning process itself [28]. In this setting, often referred to as assessment-of-learning, assessment primarily takes a summative shape [29]. In the past decades, medical educators have moved away from assessment-of-learning toward training settings that include multiple low-stake assessments and rich feedback integrated within the learning process. This environment, referred to as assessment-for-learning [30–33], stimulates formative forms of assessment [29]. However, not all assessment can be exclusively formative because at some point medical educators need to decide on trainees’ readiness to progress [29]. For this reason, hybrid approaches have been developed that combine formative and summative forms of assessment [34].

Hybrid approaches have also landed in the field of progress testing. For example, the progress test that is practiced by a consortium of Dutch medical schools consists of a series of 4 formative tests per annum which are translated into a summative end-of-year decision [18]. Another progress test, taken semi-annually in a 4-year training program in a dental school in the UK, includes formative tests in training years 1–2 and summative tests in training years 3–4 [35]. This format resembles the current DRPT format in which progress tests are formative in the first half of residency and summative in the second half. A disadvantage of hybrid testing is the difficulty to untie the separate effects of formative and summative assessments. Whereas formative progress testing has proven its potential to induce beneficial educational effects, for example, on test scores in graduates’ licensing examinations [36], the literature on learning effects of summative progress testing is limited, although this type of testing is increasingly used in residency programs [11, 12].

The extent to which a summative format in progress testing affects learning likely depends on local educational settings and regulations [18, 23, 35]. In our educational setting, we observed an overall steady performance of our summative resident group after summative progress testing was introduced, both over the course of tests and compared with the formative resident group. Although we found that relative test scores in the summative resident group differed significantly across separate DRPT tests in several PGYs, scores fluctuated across tests without any clear positive or negative trend. Therefore, even though differences across tests were statistically significant, we do not consider them relevant from an educational point-of-view and we conclude that overall group performance was steady during our observation period. The lack of a clear positive educational effect of transitioning may be unsatisfactory if summative progress testing is introduced to promote learning [18, 19]. However, it should be emphasized that the present study focuses on whole group effects. We do not exclude that the summative DRPT format may have positive educational effects in small subgroups of residents. For example, Fig. 3 suggests that after PGY 3 summative progress testing is associated with fewer outliers in the lower extreme of test scores than formative testing. This may indicate that summative progress testing stimulates learning in senior residents who reside in the lower end of test scores, which would actually align with the purpose of the program’s competency-based philosophy, i.e., to decrease the number of graduates not meeting minimum standards. This needs further research in the coming years, when the number of senior residents in the summative group is expected to increase.

Recently, Heeneman et al. compared formative and summative progress test formats in medical students [23]. They found that test scores were higher in students who followed a 4-year graduate-entry Master program with formative progress tests that were embedded in a comprehensive program of assessment than in students in a 6-year Bachelor-Master program where individual progress tests had a summative pass/fail decision. It remained speculative whether these higher scores were caused by the formative test format, the comprehensive assessment program, or the substantially different characteristics of both investigated student groups. Our results are not in line with Heeneman’s study, since we found no systematic difference in test results between summatively and formatively tested participants. We can only speculate on explanations why test results were similar in our 2 resident groups. It is known that trainees may perceive formative assessment as summative [37–39]. If this applied to the residents in our formative group, it is not surprising that they scored similar to residents in the summative group. Another explanation may be that our summative DRPT regulations were not a strong enough stimulus to induce additional beneficial educational effects in our summative resident group. A previous study showed that the summative DRPT criterion did not evoke much consent nor resistance among residents [19]. A neutral attitude to summative regulations may prevent participants to intensify their study behavior. A third explanation for the similar test results in our resident groups may be that transitioning to summative progress testing takes a longer time period to bear fruit than our 4-year observation period. These possible explanations need to be explored in future research.

The commonly used four-level pyramid of Miller [40] provides a useful framework for the development of clinical competence in medical trainees. The first level of competence is described by the term “knows,” framing the basic knowledge a trainee should have. The second level is summarized as “knows how,” covering the interpretation and application of knowledge. These 2 levels primarily apply to cognition and are often assessed through knowledge tests, of which the DRPT is an example. The subsequent pyramid levels of “shows how” and “does” refer to competence in clinical practice. These 2 levels apply more to skills and behavior than the first 2 pyramid levels and are generally assessed in clinical simulation and workplace settings. Well-known examples of such assessments are the Objective Structured Clinical Examination (OSCE) or Practical Examination (OSPE) in which a candidate moves along a series of structured examination stations that assess certain clinical skills, the Objective Structured Assessment of Technical Skills (OSATS) in which a trainee’s technical medical skills are assessed in a laboratory or clinical setting through an operation-specific checklist for Miller’s “shows how” level, and the Mini Clinical Evaluation Exercise (Mini-CEX) in which a trainee’s performance is assessed in real clinical workplace situations on multiple occasions and by various assessors: Millers “does” level. We found indications that residents from the summative DRPT group performed somewhat better than residents from the formative group. This raises the question whether differences in competence in a knowledge test such as the DRPT may propagate to higher levels in Miller’s pyramid, that is, to clinical (simulation) settings. That would align with the notion that much of the radiologist’s work happens behind a computer screen, not unlike the DRTP test arrangement. Another question that comes up is whether transitioning from formative to summative assessment has educational effects in clinical simulation and workplace settings in radiology. Whereas our study considered Miller’s first 2 competence levels, possible educational effects in the next 2 levels remain to be explored further in radiology. These questions may be addressed in future research.

This study has a number of limitations. First, we did not randomize between summative and formative progress testing. Therefore, we cannot exclude that other factors, such as participants’ characteristics and type of residency program, had an influence on resident test results. Although randomization would have given a more ideal research setting, this was not achievable because residents were restricted to the national training guidelines. Moreover, a randomization that confronts some residents with summative regulations and others not would obviously have raised considerable ethical objections. Second, the sample size was relatively small in our summative resident group in PGYs 4–5. Although we may have underestimated educational effects in these PGYs because of the small sample size, we believe the number of residents in other PGYs was sufficiently large to study the effect of transitioning to summative progress testing. But again, we cannot exclude that the residents who chose to be tested under the summative regime were different from those who chose to remain under the formative regime. Future research should show how the distribution of scores will compare between all-formative and all-summative cohorts. Third, test difficulty may have differed between separate tests. If test difficulty has increased gradually from tests #1 to #7, this may have neutralized a gradual knowledge increase in residents, leading to rather stable group results over the course of tests. However, we have no indications that the DRPT’s Examination Committee has increased test difficulty over time, other than by decreasing item chance success sum score from tests #2 to #7 (see Table 1). We have accounted for this factor by calculating relative test scores. Moreover, in our comparisons between the summative and formative resident groups, changes in test difficulty would have touched both groups.

## Conclusions

In our experience, postgraduate formative progress testing can be transitioned to a summative format without negative effects on test results of the whole resident group. To identify possible positive educational effects of transitioning to summative progress testing, we suggest that focus should be put on the subgroup of residents who reside in the lower end of test scores.
